# Enzyme Encapsulation by Facile Self-Assembly Silica-Modified Magnetic Nanoparticles for Glucose Monitoring in Urine

**DOI:** 10.3390/pharmaceutics14061154

**Published:** 2022-05-28

**Authors:** Zhimin Luo, Guoning Chen, Ke Yang, Lu Wang, Xia Cui, Jiameng Xu, Qiang Fu

**Affiliations:** 1 Department of Pharmaceutical Analysis, School of Pharmacy, Xi’an Jiaotong University, Xi’an 710061, China; luozm0905@xjtu.edu.cn (Z.L.); 18292187525@163.com (K.Y.); wanglu0914@stu.xjtu.edu.cn (L.W.); cuixia0415@stu.xjtu.edu.cn (X.C.); xujm2019@stu.xjtu.edu.cn (J.X.); 2 Department of Pharmaceutical Analysis, School of Pharmacy, Key Laboratory of Ningxia Ethnomedicine Modernization, Ministry of Education, Ningxia Medical University, Yinchuan 750004, China; 3 Department of Pharmaceutical Analysis, College of Pharmacy, Shenzhen Technology University, Shenzhen 518118, China

**Keywords:** enzyme encapsulation, glucose oxidase, colorimetric sensing method, glucose

## Abstract

Silica nanoparticles hold tremendous potential for the encapsulation of enzymes. However, aqueous alcohol solutions and catalysts are prerequisites for the production of silica nanoparticles, which are too harsh for maintaining the enzyme activity. Herein, a procedure without any organic solvents and catalysts (acidic or alkaline) is developed for the synthesis of silica-encapsulated glucose-oxidase-coated magnetic nanoparticles by a facile self-assembly route, avoiding damage of the enzyme structure in the reaction system. The encapsulated enzyme was characterized by scanning electron microscopy, transmission electron microscopy, energy-dispersive spectrometry, and a vibrating sample magnetometer. Finally, a colorimetric sensing method was developed for the detection of glucose in urine samples based on the encapsulated glucose oxidase and a hydrogen peroxide test strip. The method exhibited a good linear performance in the concentration range of 20~160 μg mL^−1^ and good recoveries ranging from 94.3 to 118.0%. This work proves that the self-assembly method could be employed to encapsulate glucose oxidase into silica-coated magnetic particles. The developed colorimetric sensing method shows high sensitivity, which will provide a promising tool for the detection of glucose and the monitoring of diabetes.

## 1. Introduction

Diabetes mellitus, one of the most widespread chronic diseases, can cause hyperglycemia and other related metabolic disturbances [1]. Many complications such as retinopathy [2], nephropathy [3], cardiovascular diseases [4], and dementia [5] are associated with the disorder of glucose metabolism. Blood glucose monitoring is an important part of diabetes management. Current monitoring of the glucose level is predominantly performed with venous blood or finger-pricking. This invasive method can cause considerable distress to the patient. Previous research has shown that glucose in urine may be a precursor of diabetes. Especially in diabetic patients, the urine glucose level is directly proportional to the level of blood glucose [6]. Thus, the concentration of glucose in urine is also a practicable indicator for screening and monitoring of diabetes. More importantly, measuring glucose in urine is non-invasive.

In general, glucose concentration has been measured by using conventional chromatography [7], capillary electrophoresis [8], liquid chromatography/tandem mass spectrometry (LC-MS/MS) [9], fluorometry [10], colorimetry [11], chemiluminescence [12], and electrochemistry methods [13]. Each of the aforementioned methods has its advantages and limitations. For example, LC-MS/MS and capillary electrophoresis show high sensitivity, but they require expensive instrumentation and expertise for operation. Electrochemical methods usually require sophisticated modification of the working electrode. Colorimetric sensors exhibit low detection sensitivity but are still popular because of the merits of low cost, rapidness, high throughput, and simplicity. In particular, colorimetric strips provide a promising platform for biochemical assays. 

Recently, various sensing methods for H_2_O_2_ have been employed to detect glucose because glucose can cause a rapid release of H_2_O_2_ by the catalytic action of its oxidases [14,15,16]. However, some inherent deficiencies of natural enzymes, such as low operational stability, low anti-interference to circumstances, and poor recyclability, limit their practical applications. Enzyme immobilization is one of the most promising methods to circumvent these problems [17,18]. A variety of materials including silica composites, magnetic material, metal–organic frameworks, and membranes are used to immobilize free enzymes. Silica nanoparticles have been utilized to protect encapsulated enzymes under harsh conditions due to their good biocompatibility, high chemical stability, low toxicity, high mechanical stability, facile controllability, and flexible surface chemistry [19,20]. Most methods for the production of silica nanoparticles were originated by Stöber et al. [21]. However, these methods are too harsh for the encapsulation of biomacromolecules because organic solvents and acid or alkaline are involved in the process of silica nanoparticles production. To date, two practicable approaches have been proposed to incorporate enzymes in silica nanoparticles. One was developed by Ellerby et al. [22]. In the reaction procedure, sonication was performed before the addition of the biomacromolecules, thus avoiding the requirement of organic solvents. Another modification for this approach was to add the biomacromolecules in a biologically compatible solution, which was achieved through the addition of buffer to increase the pH value after tetramethoxysilane underwent acid-catalyzed hydrolysis. Although some biomacromolecules could be encapsulated into a silica matrix successfully, some problems still need to be overcome, such as the difficulty of encapsulation and the insurmountable escape of negatively charged proteins from the silica matrix [23,24]. Another approach, proposed by our group, is a one-step self-assembly process in which biomacromolecules could be encapsulated in silica nanoparticles without any organic solvent and the catalyzer of acid or base [25]. The biomacromolecules can retain their entire structure and biological activity in the production process. However, the general applicability of this procedure for the encapsulation of biomacromolecules still needs to be explored by more researchers.

**Figure 1:**
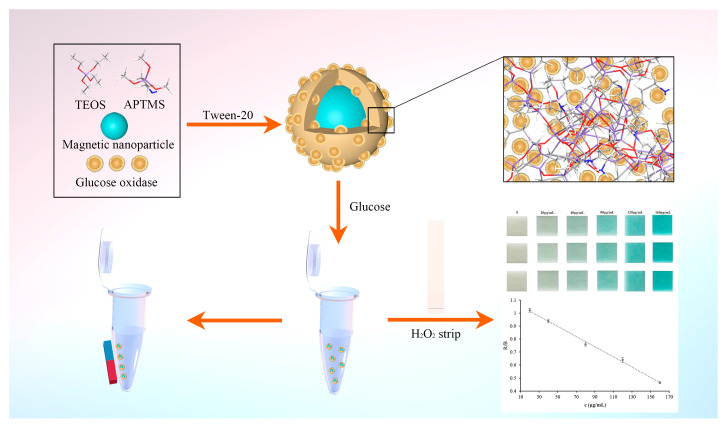
Schematic of preparation and utilization of magnetic embedded enzymes.

In this paper, we explicitly demonstrate a procedure to encapsulate glucose oxidase into silica-modified magnetic nanoparticles using a facile self-assembly route without an organic solvent and any acidic or alkaline catalysts. The introduction of magnetic nanoparticles achieves rapid separation and recycling of enzymes. The self-assembly method ensures that the enzyme retains its structure and activity. We further propose a colorimetric sensing method for the determination of glucose in urine based on the encapsulated glucose oxidase and a hydrogen peroxide test strip. In order to achieve quantitative determination of glucose by using the colorimetric sensing method, the signal was recorded using a common smartphone, and subsequently, the RGB color values were obtained using Photoshop software. The principle and procedure of this work are shown in Figure 1.

## 2. Materials and Methods

### 2.1. Materials and Reagents

FeCl_3_·6H_2_O citric acid and glucose were purchased from Tianjin Kermel Chemical Reagent Co., Ltd. (Tianjin, China). Glucose oxidase (GOx) was purchased from Shanghai Yuanye Bio-Technology Co., Ltd. (Shanghai, China). Tetraethoxysilane (TEOS) and 3,3′,5,5′-tetramethylbenzidine (TMB) were purchased from J&K Scientific, Ltd. Fluorescein isothiocyanate (FITC) and 3-aminopropyltrimethoxysilane (APTMS) were supplied by Shanghai Aladdin Biochemical Technology Co., Ltd. (Shanghai, China). Horseradish peroxidase (HRP, >300 U/mg) was obtained from McLean Biochemical Reagent, Ltd. (Shanghai, China). Hydrogen peroxide test strips were supplied by Lohand biological (Jiuhuan, China). Ethylene glycol was supplied by Tianjin Fuyu Fine Chemical (Tianjin, China). Ultrapure water was purified by Molelement 1810b. All other chemicals were analytical reagent grade and provided by local suppliers.

### 2.2. Preparation of Glucose Oxidase Embedded into Silica Nanoparticles

The Fe_3_O_4_ nanoparticles were synthesized according to our previous work [26]. FeCl_3_·6H_2_O (5.328 g) was dissolved in 160 mL of ethylene glycol under stirring, and then, 14.4 g of anhydrous sodium acetate and 5.328 g of polyethylene glycol (4000) were added into the clear and transparent solution. Then, the mixture was stirred vigorously for 1 h and transferred into two Teflon-lined stainless steel autoclaves equally. The autoclaves were placed in a heating box for the reaction at 200 °C for 12 h. After that, the mixture was cooled to room temperature. The resultant products were washed several times with ultra-pure water and ethanol, collected with an external magnetic field, and dried at 50 °C. 

The citric-acid-modified Fe_3_O_4_ particles were obtained by a reported method with some minor modifications [27]. Fe_3_O_4_ particles (200 mg) were dispersed into 200 mL deionized water and dispersed uniformly by ultrasonication. Then, 2 mL of citric acid solution (2M) was added and the reaction temperature was raised up to 90 °C. The reaction was completed for 90 min with continuous stirring. Lastly, the products were washed several times with deionized water.

Entrapped glucose oxidase was prepared as follows: 2.5 mg of citric-acid-modified Fe_3_O_4_ particles were dispersed into 0.02% Tween-20 aqueous solution (4 mL), and then, 1 mg of glucose oxidase was added, which was followed by placing APTMS (2.5 μL) and TEOS (10 μL) into this solution. The mixture was shaken at room temperature with an oscillation velocity of 150 rpm after vigorous shaking by hand. After 2 h, the resultant products were collected by magnet, washed with deionized water three times to remove unreacted reagents, and then stored in PBS at 4 °C. Entrapped FITC-labeled glucose oxidase was prepared using the same procedure mentioned above in the presence of FITC-labeled glucose oxidase.

### 2.3. Characterization

Scanning electron microscopy (SEM) images were obtained with a GeminiSEM 500. Dried samples were placed on the conductive adhesive and sprayed with gold. Transmission electron microscopy (TEM) was performed on an H-7650 device to observe the size and morphology of the samples. The surface element composition of the samples was obtained using an Oxford energy-dispersive spectrometer (EDS). The magnetic property of the samples was measured using an MPMS-SQUID VSM-094 vibrating sample magnetometer (VSM). Fluorescence images of samples were obtained using a Leica TCS SP8 STED 3X confocal laser scanning microscope (CLSM). A 6100 X-ray diffractometer (XRD) was used to characterize the crystal structure of entrapped glucose oxidase. Thermal gravimetric analysis (TGA) of entrapped glucose oxidase was performed with an SDTQ600 thermal gravimetric analyzer (New Castle, DE, USA). 

### 2.4. Activity Assay

The activity of glucose oxidase was measured using the methods of a previous report with some modification [16]. Glucose causes a rapid release of H_2_O_2_ by the catalytic action of glucose oxidase. The amount of H_2_O_2_ produced from glucose oxidation was measured based on the oxidation reaction of 3,3′,5,5′-tetramethylbenzidine (TMB) in the presence of horseradish peroxidase, accompanied with a color change from colorless to blue. Briefly, certain amounts of samples were mixed with 100 μL substrate solution (1 mg mL^−1^) at 37 °C; after 30 min, TMB solution (100 μL) and 10 μL of HRP solution (1 μg mL^−1^) were added. The reaction was continued for 10 min at 37 °C; then, 50 μL termination solution (2 M H_2_SO_4_) was added to terminate the reaction. The absorbance of the mixture was recorded using a Thermo Scientific Microplate Reader. 

The activity recovery of enzymes for embedded enzymes was determined using the following formula: Activity recovery (%) = (Total activity in embedded enzymes)/Total activity used for the embedded enzyme preparation) × 100%.

### 2.5. Evaluation of the Embedded Glucose Oxidase

The enzyme’s kinetic parameter, in the form of a Michaelis-Menten constant, was obtained according to the Michaelis–Menten equation [28]: 1/*v* = (*K*_M_/*V*_max_) × (1/[*S*]) + 1/*V*_max_, where *ν* is the enzymatic reaction rate, *V*_max_ is the maximal reaction velocity, *K*_M_ is the Michaelis–Menten constant, and [*S*] is the concentration of glucose. The Michaelis–Menten constant of the embedded glucose oxidase was determined by measuring the initial reaction rates with glucose at 37 °C for 10 min. The Michaelis–Menten constant was calculated using double reciprocal Lineweaver–Burk plots. As a control, the affinity of free enzyme was also tested under the same conditions.

The stability of the embedded enzymes was evaluated under different conditions. The enzymes were incubated under various extreme conditions (pH and temperature) for 1 h before detection. The residual activity was measured using the abovementioned activity assay method. The free enzyme was also evaluated under the same conditions as a control. The percentage of residual activity at each pH value was calculated by taking the activity at pH 7 as 100%. The percentage of residual activity at each temperature was calculated by taking the activity at 4 °C as 100%.

The reusability of the embedded enzymes was determined according to the enzyme activity and repeated seven times. After each cycle, the embedded enzymes were washed with buffer solution. The activity of the embedded enzymes obtained in the first cycle was defined as 100%.

### 2.6. Measurement Procedure for Glucose

The glucose was measured using hydrogen peroxide test strips. Firstly, the embedded glucose oxidase was suspended in buffer solution to react with different concentrations of glucose solution at 37 °C for 15 min. Glucose oxidase catalyzed the oxidation reaction of glucose for the generation of H_2_O_2_. Secondly, the hydrogen peroxide test strip was placed in the solution with H_2_O_2_ produced by the catalytic oxidation reaction. The strip was taken out of the solution after 2 s and the water from the surface of the strip was blotted up. Colorimetric analysis was then carried out by determining the RGB color values of the hydrogen peroxide test strips after exposure to various samples. The color of the hydrogen peroxide test strips was photographed using a smartphone, and the images were analyzed using Adobe Photoshop software to evaluate R, G, and B values.

### 2.7. Interference Study

Six different interfering solutions containing glycine, mannitol, sarcosine, creatinine, vitamin C, and sucrose in the buffer solution were used to study the effect of the presence of possible interfering species commonly presented in biological samples. The individual concentration of the species in the interfering solutions was 160 μg mL^−1^. All the test solutions were analyzed by the same strip procedure as glucose.

### 2.8. Assay of Glucose in Urine

Colorimetric strips were used for the quantification of glucose presented in urine samples. The urine samples, obtained from the First Affiliated Hospital of Xi’an Jiaotong University (Xi’an, China), were diluted with buffer solution according to the normal level of glucose in urine samples before detection. Three concentration levels of spiked urine samples were used to evaluate the accuracy and precision of the developed method. The measurements were performed using the same previously mentioned assay procedure of glucose. To further assess the accuracy of the assay, the concentration of glucose in three simulated positive samples was determine by the established strip method and clinical method.

## 3. Results and Discussion

### 3.1. Preparation of the Embedded Glucose Oxidase

Silica nanoparticles can be obtained by a self-condensation method under mild conditions, which has been proved by our previous work [25]. The method is more benign since no organic solvents and catalytic agents participate in the polymerization. This method has strong potential for the encapsulation of biomacromolecules. In order to exhibit the application of the method for the encapsulation of biomacromolecules, silica nanoparticles encapsulating glucose oxidase were obtained in the presence of surfactants (Tween-20) and silica sources (TEOS and APTMS) at room temperature. To achieve efficient and rapid separation and recycling of embedded enzymes, one very feasible method is the introduction of magnetic nanoparticles. In this study, magnetic Fe_3_O_4_ nanoparticles, modified by citric acid, were chosen as magnetic sources to enhance the binding affinity between silica nanoparticles and magnetic nanoparticles. The activity of the encapsulated glucose oxidase was evaluated by the catalytic oxidation of TMB in the presence of H_2_O_2_, which originated from the oxidation reaction products of glucose. The enzymatic activity could be evaluated by detecting the absorption peaks at 450 nm and terminated by H_2_SO_4_.

**Figure 2:**
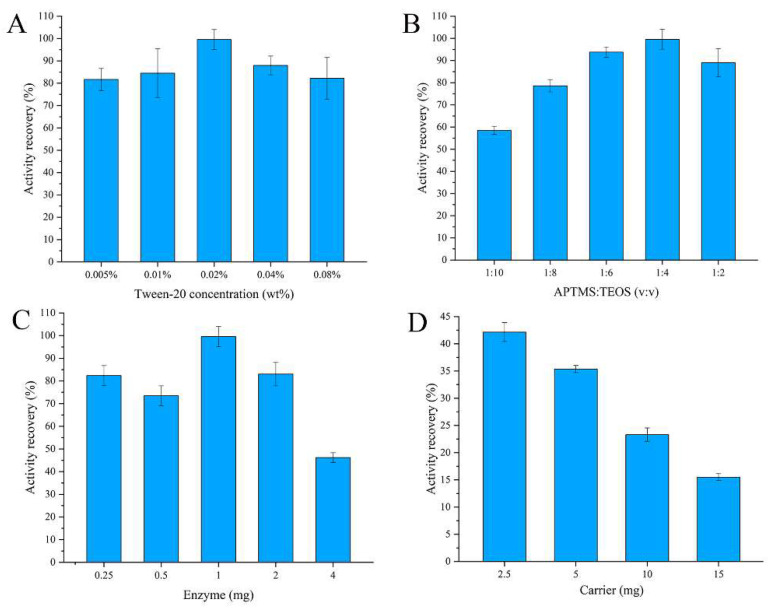
Effect of the preparation of encapsulated glucose oxidase: (**A**) concentration of Tween-20; (**B**) ratio of APTMS and TEOS; (**C**) amount of enzyme; (**D**) amount of carrier.

Several parameters such as the concentration of surfactant, the monomer ratios, the amount of enzyme, and the amount of carrier were optimized in this study. The results are shown in Figure 2. Figure 2A shows the resulting effect of concentration of the surfactant on activity recovery of the encapsulated enzyme, and it can be observed that the surfactant showed almost no influence on the activity recovery. When the monomer ratio of APTMS:TEOS was 1:4, it presented the best recovery of enzyme activity. With the decrease in the proportion of APTMS, the enzyme activity recovery was reduced because of the reduction in primary amine groups on nanoparticles. A higher activity recovery was observed when the amount of enzyme for entrapment was 1 mg. However, when exceeding this amount, the enzyme activity recovery was decreased because the enzyme could not be fully encapsulated in the silica nanoparticles. Additionally, the enzyme would be overly enwrapped when the amount of enzyme was lower than 1 mg; thus, the enzyme activity recovery was decreased. As shown in Figure 2D, the enzyme activity recovery was reduced when the carrier (citric-acid-modified Fe_3_O_4_ particles) content was increased. The cause of this phenomenon may be ascribed to the competitive combination of the carboxyl group on the surface of the carrier with amino groups in the enzyme. Overall, the results show that the optimum technological conditions are 0.02% surfactant, 2.5 mg of carrier, 1 mg of enzyme, and a ratio of 1:4 of APTMS:TEOS.

### 3.2. Evaluation of Magnetic Embedded Enzymes

The catalytic kinetic parameters of the embedded glucose oxidase were further investigated. As shown in Figure S1, the plots of initial velocity versus glucose concentration followed typical Michaelis–Menten behaviors. The Michaelis–Menten constant was calculated using Lineweaver–Burk plots of double reciprocal of the Michaelis–Menten equation: 1/*ν*  =  *K*_M_/*V*_max_ (1/[*S*]  +  1/*K*_M_), where *ν* is the initial velocity, *K*_M_ is the Michaelis–Menten constant, *V*_max_ represents the maximal reaction velocity, and [*S*] corresponds to substrate concentration of glucose. The *K*_M_ and *V*_max_ values for glucose were 18.15 mM and 2.25 mM min^−1^, respectively, which were calculated using the double reciprocal curve. Table S1 lists the *K*_M_ value in this work and other previously reported results [29,30,31,32,33]. It proves that the embedded glucose oxidase retained relatively good affinity to the substrate of glucose.

**Figure 3:**
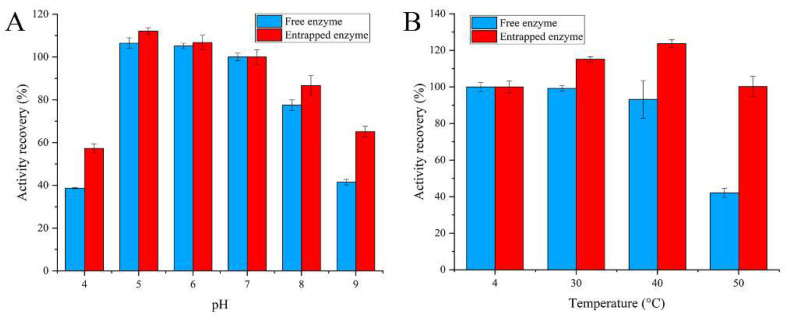
Stability of entrapped glucose oxidase: (**A**) stability of pH; (**B**) stability of temperature.

The encapsulation procedure for enzymes generally has a protective effect on the activity of the enzymes. The residual activity of the enzymes was investigated after exposing them to some extreme environments to evaluate the protection effect. The pH value is one of the most important parameters that affects the activity of enzymes. As seen in Figure 3A, high stability was obtained for both embedded and free enzymes when the pH values were 5~7. The enzyme activity of the embedded enzyme and free enzyme gradually decreased when the pH value was higher than eight or lower than five. However, in general, the embedded enzyme demonstrated greater pH stability than the free form under the same conditions. Figure 3B shows the influence of temperature on the stability of the embedded enzyme. The activities of the embedded enzyme and free enzyme showed no reduction when the temperature was lower than 40 °C. The embedded enzyme still retained about 100% of its initial activity at 50 °C, whereas the free enzyme retained only 40% of its initial activity. These results show that silica nanoparticles significantly enhance the resistance of glucose oxidase to thermal inactivation.

**Figure 4:**
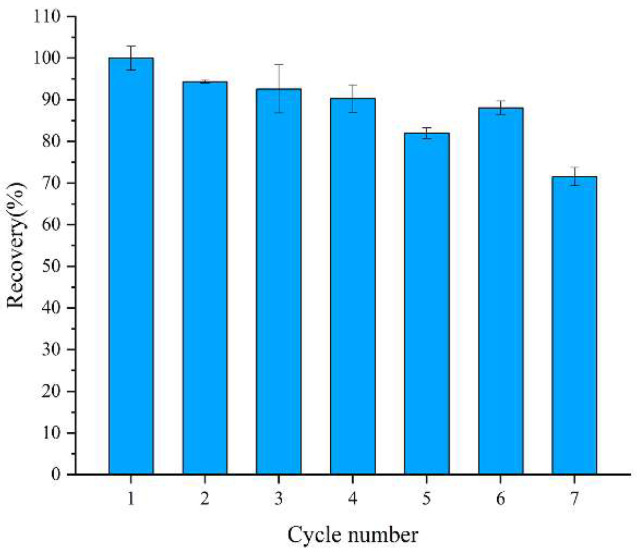
Recyclability of encapsulated glucose oxidase.

The reusability of embedded enzymes is an important evaluating indicator in practical applications. The residual activity utilization of the embedded enzymes was demonstrated for up to seven cycles. As shown in Figure 4, the enzymatic activity decreased after almost every cycle, which was ascribed to the incomplete elution of the substrate and the leakage of enzyme from the carrier. However, the residual activity of the embedded enzymes still reached about 75% of the initial activity after seven reuses. The results illustrate that the embedded enzymes have high reusability.

### 3.3. Characterization of Magnetic Embedded Enzymes

**Figure 5:**
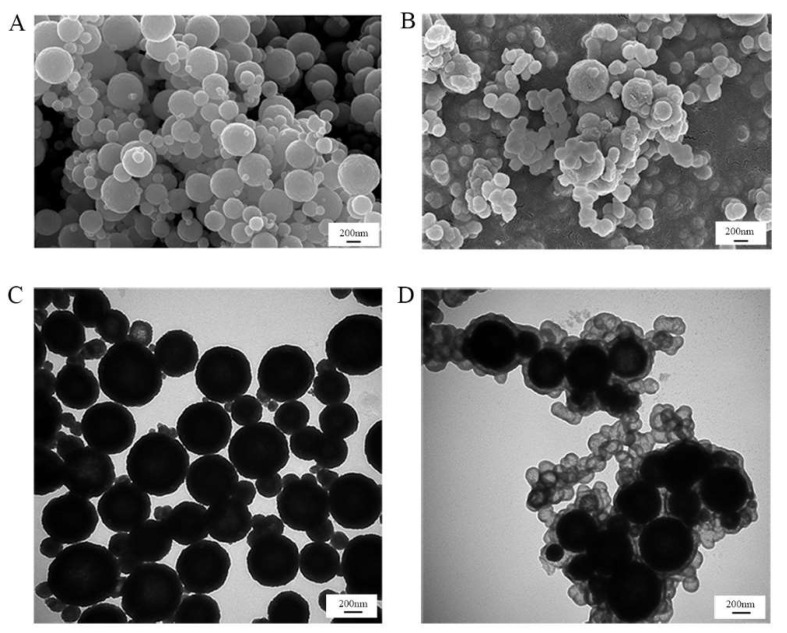
SEM and TEM images of Fe_3_O_4_ (**A**,**C**) and encapsulated glucose oxidase (**B**,**D**).

In order to better observe the successful combination of silica nanoparticles and Fe_3_O_4_ nanoparticles, the morphologies of silica-coated Fe_3_O_4_ nanoparticles were characterized by SEM and TEM. As shown in Figure 5, the average size of the Fe_3_O_4_ nanoparticles was 300~400 nm, and the silica layer or small silica nanoparticles were tightly entrapped onto the surface of the Fe_3_O_4_ nanoparticles. The energy-dispersive spectrometry (EDS) results are shown in Figure S2A,B, demonstrating the existence of C, O, and Fe in the magnetic nanoparticles. Besides C, O, and Fe, N and Si were present in the silica-coated magnetic nanoparticles, verifying the successful generation of silica nanoparticles on the surface of magnetic nanoparticles.

**Figure 6:**
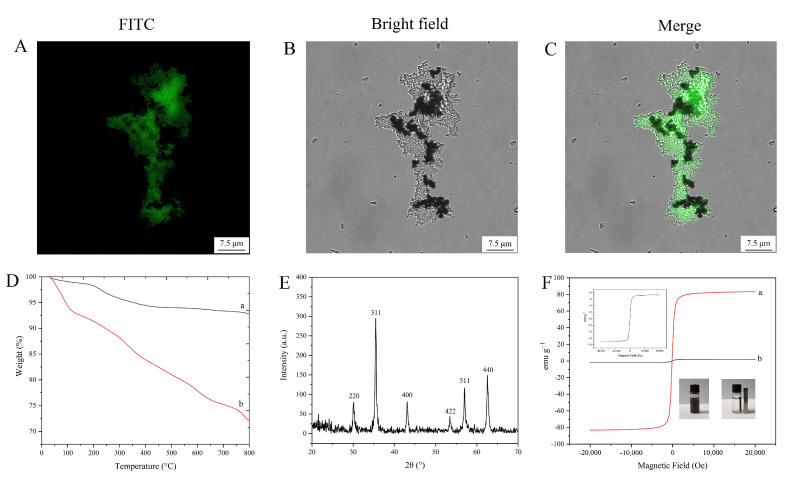
Characterization results: (**A**–**C**) laser confocal microscopy images; (**D**) TGA curves (a: Fe_3_O_4_, b: encapsulated glucose oxidase); (**E**) X-ray diffraction image; (**F**) magnetic hysteresis regression curves (a: Fe_3_O_4_, b: encapsulated glucose oxidase).

To further confirm that the glucose oxidase was embedded into the silica-coated magnetic nanoparticles, FITC-labeled glucose oxidase was used. The confocal laser scanning microscopy images (Figure 6A–C) indicated the successful encapsulation of FITC-labeled glucose oxidase.

TGA curves of Fe_3_O_4_ nanoparticles and magnetic embedded enzymes are shown in Figure 6D. Both of them show a lower weight loss (lower than 8.6 wt%) below 200 °C, which could be considered as the evaporation of water. When the temperature was increased to 800 °C, another weight loss (about 5.4 wt%) of Fe_3_O_4_ nanoparticles was observed, which could be ascribed to the decomposition of organic parts. However, a large mass loss (about 19.3 wt%) of the encapsulated enzyme nanoparticles was observed, which was mainly considered as the decomposition of the silica-encapsulated enzyme complexes. The results indicate that Fe_3_O_4_ nanoparticles were successfully grafted with the silica-encapsulated enzyme complex.

XRD patterns of the magnetic embedded enzymes are shown in Figure 6E. The crystals had strong peaks at 2θ of 30.22°, 35.54°, 43.14°, 53.56°, 57.02°, and 62.62°, showing no difference with Fe_3_O_4_ diffraction [34]. This result indicates that the embedding procedure for the enzyme did not affect the crystal structure of Fe_3_O_4_.

Vibrating sample magnetometry measurement (Figure 6F) indicated that the magnetic embedded enzymes exhibited a magnetic hysteresis loop with a magnetization saturation value of 1.7 emu g^−1^, revealing that the magnetic embedded enzymes had a lower saturating magnetization compared to the bare magnetic materials (83.3 emu g^−1^). However, the as-prepared magnetic composites still exhibited a strong magnetic response, which could be rapidly attracted toward a permanent magnet (inset of Figure 6F).

### 3.4. Assay of Glucose

**Figure 7:**
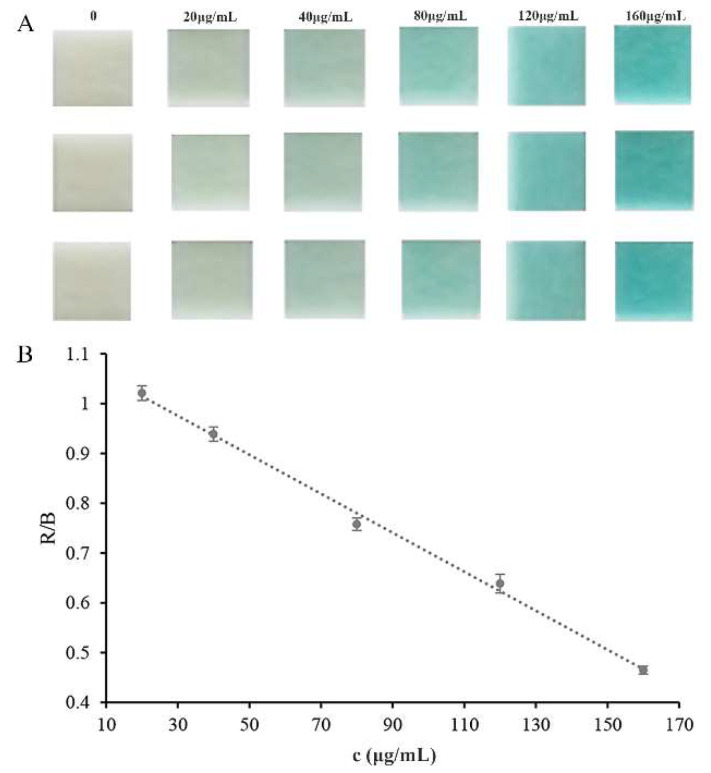
Calibration curve for glucose detection: (**A**) color results; (**B**) calibration curve results. R (red) and B (blue) are RGB color values.

Glucose could cause a rapid release of H_2_O_2_ by the catalytic action of embedded glucose oxidase. Hydrogen peroxide test strips were used to assess the response to the released H_2_O_2_ and exhibited color changes from colorless to blue. The color change could be easily detected visually, without any sophisticated or expensive instruments. Photoshop software was used to monitor the test strips’ color and extract the color alterations (RGB) after the addition of glucose, and the concentration of glucose in the solution could easily be quantified. As shown in Figure 7A, the color of the test strip was very sensitive to the change in the glucose concentration, and the color depth increased with the increase in glucose concentration. A linear correlation of the color depth with the glucose concentration was observed in the glucose concentration ranging from 20 to 160 μg mL^−1^ (y = −0.0039x + 1.0929, R^2^ = 0.9962, Figure 7B). The limit of detection (LOD), 8.8 μg mL^−1^, was calculated as three times the standard deviation of the blank signals divided by the slope of the calibration curve. The developed assay method based on the embedded enzymes and hydrogen peroxide test strips was compared with other reported methods [10,35,36,37,38]. The results are listed in Table 1. As we all know, mass spectrometry and fluorescence detectors possess higher sensitivity. However, the colorimetric method is still promising due to its rapid and simple properties. Moreover, our method exhibits superior sensitivity to commercial test strips.

### 3.5. Interference Experiments

**Figure 8:**
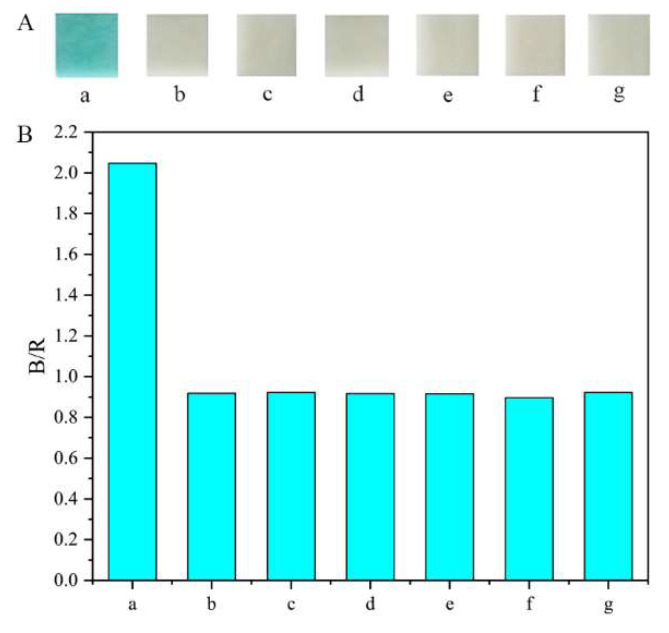
Selectivity of the developed method for glucose: (**A**) color results; (**B**) statistical results. a: glucose; b: glycine; c: mannitol; d: sarcosine; e: creatinine; f: vitamin C; g: sucrose.

To evaluate the specificity of the method, a series of interfering chemicals, including glycine, mannitol, sarcosine, creatinine, vitamin C, and sucrose, were assessed, and their concentration was the same as that of glucose (160 μg mL^−1^). As shown in Figure 8, the relative signal change caused by glucose could be well discriminated from those of other interferences. The high specificity of the method can be attributed to the strong affinity between glucose oxidase and glucose.

### 3.6. Assay of Glucose in Urine Samples

In a practical application, our strip method was used to detect the content of glucose in human urine samples. Spiked urine samples with three different concentration levels were assayed using the strip method to evaluate the accuracy and precision. The data are listed in Table 2. The recovery of glucose with different concentrations ranged from 94.3% to 118.0%, and the relative standard deviation (RSD) ranged from 1.7% to 3.4%. To further assess the method, the content of glucose in some simulated samples with different concentrations was determined using the established strip method and the clinical strip method. The data in Table S2 demonstrate that there was no significant difference between the developed strip method and the clinical strip method. These results indicate that the established method exhibits high credibility for the detection of glucose in real samples.

## 4. Conclusions

In this study, a procedure was developed successfully for the synthesis of magnetic embedded enzymes using a facile self-assembly route without alcohol and any acidic or alkaline catalysts in the preparation procedure. As a result, the activity of glucose oxidase was well preserved during the mild encapsulation procedure. The magnetic embedded enzymes exhibited high stability under some harsh conditions in contrast to free glucose oxidase. The magnetic embedded enzymes exhibited great magnetic properties and stability, resulting in rapid separation and efficient recycling by utilizing external magnetic fields. In addition, the magnetic embedded enzymes were employed to establish a colorimetric sensing method for the detection of glucose in urine with a hydrogen peroxide test strip. This method showed satisfactory linearity, precision, and accuracy. This work demonstrated the practicality of the detection system for glucose monitoring in urine samples. Additionally, the presented facile self-assembly protocol could be used for the encapsulation of enzyme under mild conditions.

## Figures and Tables

**Table 1:** Comparison of different methods for detection of glucose.

Detection Method	Detection Range (μg mL^−1^)	References
Fluorescent probe	9–900	[10]
Fluorescent probe	0.72–9	[35]
Colorimetric assay	18–360	[36]
Colorimetric assay	252–1260	[37]
Electrochemical sensor	1.8–255.6	[38]
Colorimetric strip	504–19,800	Commercial strip
Colorimetric strip	20–160	This work

**Table 2:** Recovery and precision of the method for the detection of glucose (*n* = 3).

Spiked (mg mL^−1^)	Found (mg mL^−1^)	Recovery (%)	RSD (%)
0	——	——	——
0.5	0.496 ± 0.017	99.2	3.4
1	0.943 ± 0.016	94.3	1.7
2	2.359 ± 0.048	118.0	2.0

## Data Availability

Not applicable.

## Supplementary Materials

The following supporting information can be downloaded at: https://www.mdpi.com/article/10.3390/pharmaceutics14061154/s1, Figure S1: Experimental results of enzymatic kinetics (n = 3): (A) enzymatic kinetic assay; (B) double-reciprocal plots; Figure S2: EDS results of immobilized glucose oxidase, (A) EDS mapping of Fe_3_O_4_; (B) EDS mapping of immobilized glucose oxidase; Table S1: Comparison of Michaelis constants (*K*_M_) of the entrapped enzyme and other reported entrapped enzymes; Table S2: Comparison of the detection results of glucose detected by this method and clinical test strip method.
