# Supplementary material for: Enzyme Encapsulation by Facile Self-Assembly Silica-Modified Magnetic Nanoparticles for Glucose Monitoring in Urine
Source: Pharmaceutics. 2022 May 28;14(6):1154. doi: 10.3390/pharmaceutics14061154 (PMC9227432; doi:10.3390/pharmaceutics14061154)
Supplement: Supplementary file 1 [file pharmaceutics-14-01154-s001.zip › pharmaceutics-1702887-supplementary.pdf]

Enzyme encapsulation by facile self-assembly silica modified magnetic nanoparticles for glucose monitoring in urine

Zhimin Luo [1], Guoning Chen [1,2,*], Ke Yang [1], Lu Wang [1], Xia Cui [1], Jiameng Xu [1] and Qiang Fu [1,3,*]

1 Department of Pharmaceutical Analysis, School of Pharmacy, Xi’an Jiaotong University, Xi’an 710061, China;

2 Department of Pharmaceutical Analysis, School of Pharmacy, Key Laboratory of Ningxia Ethnomedicine Modernization, Ministry of Education, Ningxia Medical University, Yinchuan 750004, China

3 Department of Pharmaceutical Analysis, College of Pharmacy, Shenzhen Technology University, Shenzhen 518118, China

* Correspondence: nycgn2022@163.com (G.C.); fuqiang@mail.xjtu.edu.cn (Q.F.)

List of contents

Supplemental data

Fig.S1

Fig.S2

Table S1

Table S2

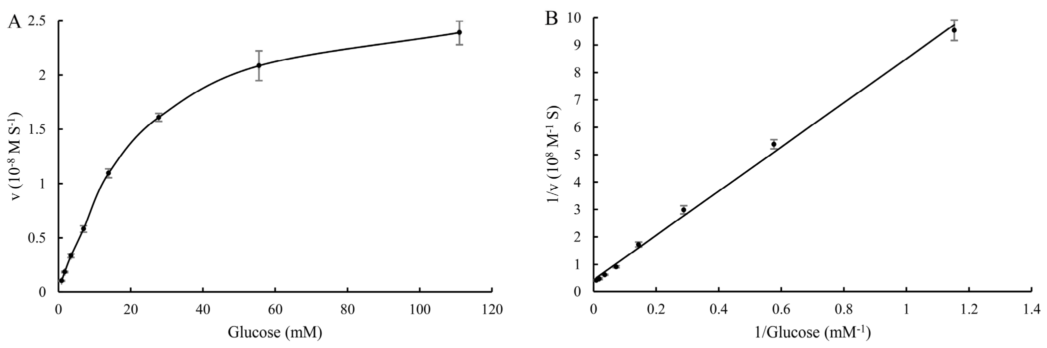

Figure S1 Experimental results of enzymatic kinetics (n=3), (A) enzymatic kinetic assay; (B) double-reciprocal plots

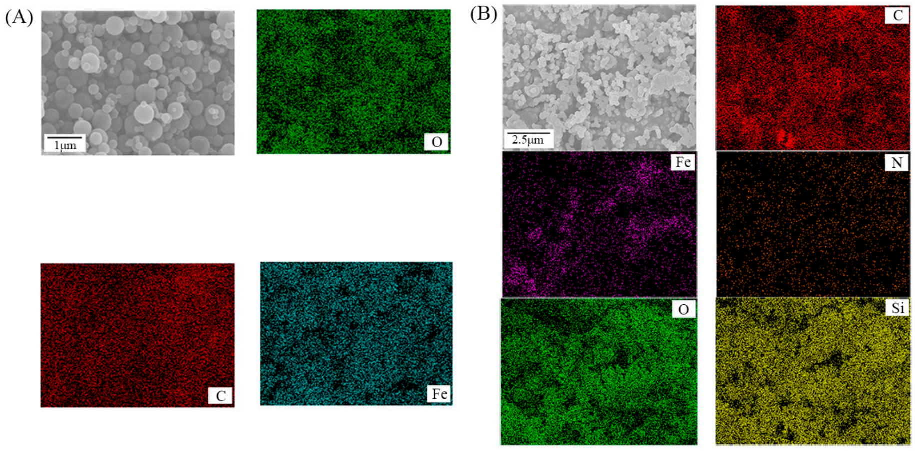

Figure S2 EDS results of immobilized glucose oxidase, (A) EDS mapping of Fe_3O_4; (B) EDS mapping of immobilized glucose oxidase

Table S1 Comparison of Michaelis constants (K_m) of the immobilized enzyme and other reported immobilized enzyme

Immobilization method	Support carrier	K_M (mM)	References
Covalent binding	Eupergit C	84.225	[29]
Covalent binding	Capillary	7.94	[30]
Adsorption	Multiwall carbon nanotubes	6.6	[31]
Covalent binding	Propylamine modified magnetite	0.69	[32]
Cross-linking method	-	12.4	[33]
Entrapment	Fe_3O_4 particles	18.15	This work

Table S2 Comparison of the detection results of glucose detected by this method and clinical test strip method

Sample	Spiked (mg mL^{-1})	Found (mg mL^{-1})	
		This method	Clinical method
Sample 1	0	No Found	No Found
Sample 2	1	0.8-1.2	+(0.5-2.5)
Sample 3	2.5	2-4	++(1-5)
Sample 4	5	4-6	+++(2.5-10)
